# Comparison and Optimization of Different Protein Nitrogen Quantitation and Residual Protein Characterization Methods in Dietary Fiber Preparations

**DOI:** 10.3389/fnut.2019.00127

**Published:** 2019-08-14

**Authors:** Felix Urbat, Patrick Müller, Andreas Hildebrand, Daniel Wefers, Mirko Bunzel

**Affiliations:** Department of Food Chemistry and Phytochemistry, Institute of Applied Biosciences, Karlsruhe Institute of Technology (KIT), Karlsruhe, Germany

**Keywords:** dietary fiber, plant cell walls, residual protein, Kjeldahl nitrogen, 6-aminoquinolyl-N-hydroxysuccinimidyl carbamate (AQC), ammonia electrode

## Abstract

Proteins are plant cell wall components but they are not included in the definition of dietary fiber. Therefore, dietary fiber preparations have to be corrected for their residual protein contents. This is commonly done by calculating the residual protein concentrations from the nitrogen contents after Kjeldahl digestion. Here, three different methods to determine nitrogen in Kjeldahl digests were compared: conventional titration with hydrochloric acid after steam distillation, a colorimetric assay (24-well microplates and cuvettes), and the determination by using an ammonia electrode. All assays gave similar results but detection using the ammonia electrode was found to be the most time-efficient approach. Also, an amino-acid profiling method, which is not based on commercial kits and which is suitable for routine analysis of dietary fiber preparations, was established. For this purpose, an HPLC-FLD method following amino acid derivatization using 6-aminoquinolyl-*N*-hydroxysuccinimidyl carbamate (AQC) was optimized for fiber samples. Although all commonly used dietary fiber preparation methods involve the application of proteases the amino acid profiles of fiber samples from different sources were shown to be quite diverse. Considering the amino acid composition of the residual protein in various dietary fiber preparations, residual protein is probably not only based on structural proteins.

## Introduction

Plant cell walls are major contributors to dietary fiber (DF) in food products and therefore an important part of human nutrition. Among others, the increased consumption of DF is associated with a reduced incidence of colon cancer, cardiovascular diseases, and metabolic disorders such as diabetes type 2 (1). DF is a complex mixture of different non-protein biopolymers and oligomers that are not degradable by human digestion enzymes but are partially fermentable by the human gut microbiota (2, 3). The international consensus definition as published in 2009 (CODEX Alimentarius definition) and comparison to former DF definitions has been discussed previously (4). Proteins have generally been excluded from the DF definition because the fermentation of non-digested proteins in the colon is rather associated with adverse health effects even though this link to gut health has not been conclusively established (5). Analytical and preparative DF isolation protocols include three consecutive enzymatic steps using the following enzymes: an α-amylase, a protease, and an amyloglucosidase. α-Amylase and amyloglucosidase digest non-resistant starch that is not included in the DF definition (2), the protease partially digests polypeptides such as enzymes, storage proteins, and structural proteins. Due to incomplete protein digestion, the obtained fiber preparations need to be corrected for the residual protein content in addition to the ash content.

Recommended analytical approaches to determine residual protein concentrations via their nitrogen contents are a combustion method (Dumas method) or Kjeldahl digestion (6, 7). However, methods to detect Kjeldahl ammonia-N are not further specified. Thus, the first aim of this study was to compare three different ammonia-N detection methods, the conventional titration with hydrochloric acid, a colorimetric assay according to Willis et al. (8) and usage of an ammonia electrode. Also, amino acid profiles of residual protein of DF preparations were in our focus because the origin of the common Kjeldahl conversion factor of 6.25 (assuming a nitrogen content of 16% in the protein) and its first adaption are basically unknown. Mariotti et al. (9) assume that it was established in the Nineteenth century. However, the nitrogen content of a protein depends on the amino acids that comprise its primary structure (9–11). Thus, the amino acid composition of a protein determines its specific conversion factor, which can easily be calculated from the nitrogen content of the contributing amino acids. The issue of using a single nitrogen-protein conversion factor and the questionable validity of this approach is not only of interest in food analysis but for agricultural and natural products in general (12, 13). For DF preparations, it was also of interest whether the residual protein is mostly made up of structural proteins, a hypothesis that has often been raised in the DF community. Thus, the second analytical aim of our study was to establish and apply an amino acid profiling method suitable for routine analysis of DF preparations.

In 1993, Cohen and Michaud discovered 6-aminoquinolyl-*N*-hydroxysuccinimidyl carbamate as a very effective pre-column derivatization reagent for primary and secondary amines (14). Hydroxyproline belongs to the latter and is—together with proline and glycine—an important marker amino acid for structural proteins (15). Because DF is mostly made up of cell wall polymers and it was hypothesized that residual protein mainly consists of structural proteins it was important to cover these three amino acids, especially the secondary amine hydroxyproline, which is formed post-translationally (16). In this work, we adapted the AQC method independently of commercial kit suppliers as previously described (17) and established it as a routine method to get a first insight into the effectiveness of the applied proteases and to examine the applicability of the historical Kjeldahl conversion factor to DF preparations. To demonstrate that the adapted method is applicable to a wide range of DF preparations, we tested it on soluble dietary fiber (SDF) and insoluble dietary fiber (IDF) preparations of pear, asparagus, buckwheat, wild rice, and wheat.

## Materials and Methods

### Chemicals and Enzymes

Enzymes used for preparative DF isolation: Heat-stable α-amylase Termamyl 120 L (from *Bacillus licheniformis*, 120 KNU g^−1^), the protease Alcalase 2.5 L (from *Bacillus licheniformis*, 2.5 AU g^−1^), and the amyloglucosidase AMG 300 L (from *Aspergillus niger*, 300 AGU g^−1^) were from Novozymes (Denmark). Enzymes used for analytical DF isolation: α-Amylase (from porcine pancreas, 16 U mg^−1^ solids) was from Sigma (USA), the amyloglucosidase (from *Aspergillus niger*, ca. 3,260 U mL^−1^, 40°C, pH 4.5, soluble starch), and the protease (Subtilisin A from *Bacillus licheniformis*, ca. 6 U mg^−1^ protein, 50 mg mL^−1^) were from Megazyme (Ireland). Amino acid standard compounds were from several suppliers (Serva, Germany; Sigma, USA; Merck, Germany; Fluka, Switzerland; Alfa Aesar, Germany; Roth, Germany) with a purity of at least 98%. 6-Aminoquinolyl-*N*-hydroxysuccinimidyl carbamate (AQC) was purchased in ready-to-use aliquots of 1 mg from Chemodex (Switzerland). Bovine serum albumin (98%) was from Roth (Germany), and the selenium catalyst mixture (according to Wieninger) from Fluka (Switzerland).

### Plant Material

Pear (*Pyrus communis* L. var. Alexander Lucas) and asparagus (*Asparagus officinalis* L.) were obtained from local suppliers. Wild rice (*Zizania aquatica* L.), buckwheat (*Fagopyrum esculentum* L.), and wheat (*Triticum aestivum* L.) were purchased in a local grocery store. Asparagus was peeled, cores of the pears were removed, and their edible tissues were lyophilized.

### Preparative Isolation of Soluble and Insoluble Dietary Fiber

All samples were ground to a particle size of < 0.5 mm. Grain flours were defatted with acetone. Sample flours were suspended in 0.08 M sodium phosphate buffer, pH 6.2 (200 mL), and 1.5 mL of α-amylase was added. Suspensions were heated in a water bath for 20 min (92°C) and gently shaken every 5 min. After cooling down the samples to room temperature in ice-water, the pH was adjusted to 7.5 with 0.275 M NaOH. Protease (600 μL) was added, and the suspensions were incubated for 30 min at 60°C under continuous agitation. The samples were cooled down, and the pH was adjusted to 4.5 with 0.325 M HCl. Amyloglucosidase (700 μL) was added, and the samples were incubated for another 30 min at 60°C. The suspensions were centrifuged, and the residues (IDF) were washed three times with water (60°C) and two times each with ethanol (99.5%, v/v) and acetone, respectively. Water fractions were combined for subsequent soluble fiber precipitation by adding the fourfold volume of ethanol (99.5%, v/v). Precipitation of the soluble fiber fractions was completed overnight. The precipitate was washed twice each with ethanol (80%, v/v), ethanol (99.5%, v/v), and acetone, respectively. Finally, the samples were stored in a hood and dried overnight at 40°C in a vacuum oven.

### Analytical Determination/Isolation of Dietary Fiber

DF was analyzed by using the official AOAC method 2009.01 according to McCleary et al. (7) with minor modifications allowing for further analysis of the fiber preparations. In brief, ground samples were suspended in 1 mL of ethanol and 40 mL of sodium maleate buffer (pH 6.0) in a 250 mL flask and were incubated with porcine pancreas α-amylase (2,000 U) and amyloglucosidase (136 U) for 16 h at 37°C. After adjusting the pH with 0.75 M Trizma base to 8.2, enzymes were inactivated by heating for 20 min at 95°C. Suspensions were incubated for another 30 min with protease (30 U) at 60°C. After adjusting the pH with 2 M acetic acid to 4.3, residues after centrifugation were washed twice with 25 mL and once with 10 mL of water (preheated to 60°C), and twice each with 25 mL of ethanol (99.5%, v/v), and acetone, respectively. After each washing step, the solvent was removed by centrifugation. The samples were stored in a hood and dried overnight at 40°C in a vacuum oven.

### Kjeldahl Digestion

Kjeldahl digestion was performed by heating samples (100–200 mg) with a selenium catalyst mixture (500 mg) in 5 mL of concentrated H_2_SO_4_ for 1.5 h in a micro Kjeldahl digestion unit. Resulting solutions were made up to 50 mL and were used for colorimetric and electrochemical assays as described below. Kjeldahl digestions for subsequent titrimetric ammonia determination started with 100–600 mg of sample and were performed in a Kjeldaltherm® block digestion unit (Gerhardt, Germany) in 15 mL of concentrated H_2_SO_4_ and one tablet (2.5 g) of Kjeldahl catalyst.

### Titrimetric Ammonia Quantitation

Steam distillation after Kjeldahl digestion was carried out in a Vapodest® 20 unit (Gerhardt, Germany). The distillate was collected in an Erlenmeyer flask containing 50 mL of 4% boric acid and Tashiro indicator. Titration was performed using 0.1 M HCl_._

### Colorimetric Ammonia Quantitation

Spectrophotometric ammonia detection was carried out according to Willis et al. (8). Sample solution (100 μL) was added to 4 mL of color reagent consisting of 3.2 g of sodium salicylate, 8.0 g of trisodium phosphate, and 50 mg of sodium nitroprusside in 100 mL of water. After adding 1 mL of 0.25% (w/v) sodium hypochlorite solution, the samples were shaken and measured 10 min later in a Jasco V-550 spectrophotometer at a wavelength of 685 nm. Calibration solutions containing nitrogen contents (from NH_4_Cl) from 12 to 44 mg N L^−1^ were treated as described for the sample solutions. H_2_SO_4_ concentration of the samples was mimicked by replacing 15 μL of the color reagent with 15 μL of 12 M H_2_SO_4_. The blank value was prepared with water instead of using sample solution (again 15 μL of the color reagent was replaced with 15 μL of 12 M H_2_SO_4_). Volumes for 24-well microplate measurements were simply downscaled: sample solutions (30 μL) were mixed with 800 μL of color reagent and 200 μL of 0.25% sodium hypochlorite solution. Again, 5 μL of 12 M H_2_SO_4_ was added to the calibration solutions and replaced the equal volume of colorant solution.

### Quantitation With an Ammonia Electrode

The diluted Kjeldahl digest (see section Kjeldahl Digestion) was further diluted 1 to 50 (v/v). An aliquot of the resulting solution (1 mL) was added to 49 mL of H_2_O in a beaker and alkalized with 3 mL of 2 M NaOH. The ammonia concentration was measured directly using an Orion™ high-performance ammonia electrode (ThermoFisher Scientific, Germany) connected to an Orion Star™ A214 pH/ISE benchtop meter. Calibration solutions containing 0.1, 1, and 10 mg of N L^−1^ (from NH_4_Cl) were freshly prepared daily. In order to mimic the ion strength of the sample solutions H_2_SO_4_ was added to the calibration solutions (1:12.5, v/v).

### Dietary Fiber Hydrolysis, AQC Derivatization, and Liquid Chromatography

Samples (100 mg) were weighed into screw cap tubes and suspended in 5 mL of half-concentrated HCl. Tubes were purged with nitrogen and subsequently heated for 20 h at 115°C. After centrifugation, solutions were filtered through a syringe filter (0.45 μm, PTFE) and made up to 50 mL with H_2_O. Aliquots of the filtrate (20 μL) and 20 μL of the internal standard solution (0.1 mM norleucine) were mixed with 180 μL of pH 8.8 borate buffer and 50 μL of AQC reagent (1 mg AQC in 333 μL of acetonitrile). Samples were heated for 10 min at 55°C and subsequently prepared for HPLC analysis. Calibration was carried out using amino acid concentrations ranging from 0.0025 to 0.2 mM. Calibration solutions were treated identically to the sample filtrates.

Liquid chromatography was performed on a ternary Shimadzu prominence system equipped with an RF-10AXL fluorescence detector using a Phenomenex Luna C18(2) column (250 mm x 4.6 mm; 5 μm). The column oven temperature was 35°C, the flow rate was held constantly at 1 mL/min, and the injection volume was 10 μL. Elution was carried out using 60 mM sodium acetate pH 6.35 (A), acetonitrile/water (60/40, v/v) (B), and water (C). The gradient started with 94% A and 6% B and changed linearly over 30 min to 87% A and 13% B. The eluent composition at 30.5 min was 80% A and 20% B and changed linearly to 76% A and 24% B after 38 min and to 73.7% A and 26.3% B after 48 min. After a flushing step with 50% B and C until 55 min, re-equilibration at 94% A and 6% B was performed for 10 min prior to the next injection. Fluorescence detection was performed using an excitation wavelength of 250 nm and an emission wavelength of 395 nm.

### Calculation of Kjeldahl Conversion Factors

The Kjeldahl conversion factors were calculated on the basis of the amino acid profile obtained from the AQC method using the following equation

(1) $$\begin{array}{r} K=\frac{1}{\sum_{k=1}^{n}\frac{a_{k}\cdot 14}{M_{k}-18}\cdot q_{k}} \end{array}$$

where *K* is the Kjeldahl conversion factor, *n* is the number of amino acids, *k* is the amino acid, *a*_*k*_ is the number of nitrogen atoms in amino acid *k, M*_*k*_ is the molecular weight of amino acid *k* and *q*_*k*_ is the fraction of amino acid *k* in the amino acid profile. The formula is based on the assumption that the amino acid chains are indefinitely long. Therefore, for every amino acid the equivalent mass of a water molecule (18 g mol^−1^) is subtracted from the molecular weight. As proteins generally contain 50–2,000 amino acids the accurate value would be between 17.64 and 17.99 g mol^−1^. However, the resulting deviation is not relevant for our purposes.

### Statistics

All statistical analyses were conducted in R (18). For the one-way analysis of variance (ANOVA) and the Tukey *post-hoc* test the commands *aov* and *TukeyHSD* were used, respectively.

## Results and Discussion

### Nitrogen Detection After Kjeldahl Digestion

**Figure 1:**
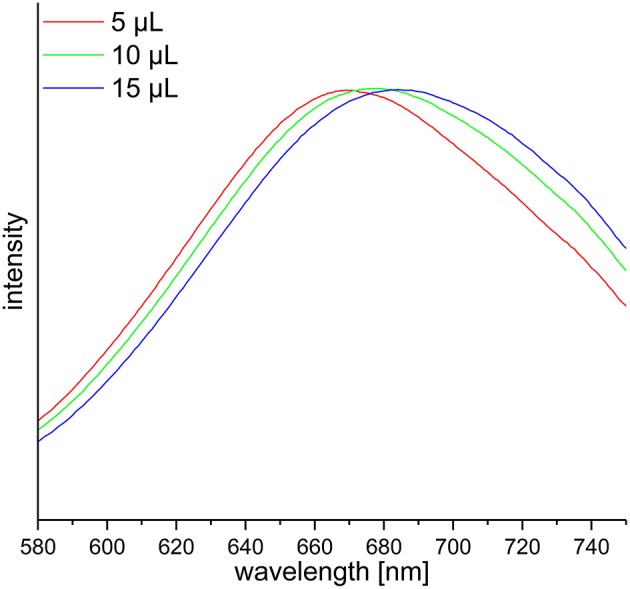
Vis-spectra of three calibration solutions used for the spectrophotometric determination of ammonia according to Willis et al. (8) with different H_2_SO_4_ adjustments in the cuvette. The absorption maxima are in ascending order 670, 677, and 685 nm.

DF preparations were obtained from a preparative procedure as described in section Preparative Isolation of Soluble and Insoluble Dietary Fiber. Three different methods to determine nitrogen after Kjeldahl digestion were applied to these samples, compared and optimized. The official AOAC method (2009.01) for the determination of total DF combining both an enzymatic-gravimetric approach and liquid chromatography recommends the application of either Kjeldahl analysis or a combustion method to quantitate residual protein (6, 7). However, nitrogen (or more specifically ammonia/ammonium) analysis after Kjeldahl digestion is not further specified. Besides titration, Kjeldahl nitrogen can be analyzed spectrophotometrically or by using an ammonia electrode. The latter procedure has been demonstrated in the past for various biological material including, for example, wort, beer, soil samples, wood bark, and other plant material (19–22). Both ammonia detection by using an ammonia electrode and by using the spectrophotometric method according to Willis et al. (8) needed to be optimized because initial experiments gave inaccurate results in recovery experiments with bovine serum albumin (data not shown). According to Willis et al. (8) the calibration solutions can be treated just as the sample solutions to form the ammonia dependent dye. However, the absorption spectrum of the resulting dye shifts depending on the H_2_SO_4_ concentration in the test solution (Figure 1). Accurate results are achieved by replacing 15 μL of the coloring reagent with H_2_SO_4_ for the calibration solutions. Also, calibration solutions for nitrogen detection using an ammonia electrode had to be adjusted for the H_2_SO_4_ concentration of the sample solutions because the response of the electrode is dependent on the ionic strength of the solution to be measured. The adjustment assumes that after Kjeldahl digestion 4 out of 5 mL of the concentrated H_2_SO_4_ remain in the Kjeldahl flask. As shown in Table 1 all nitrogen detection methods that were tested in this study show comparable results and standard deviations. An exception are the slightly higher standard deviations for the down-scaled (24-well plate) version of the colorimetric approach, which may be due to smaller pipetting volumes. One-way ANOVA showed differences for three out of ten samples analyzed (α > 0.01). In these cases, the differing pairs were determined using Tukey's test. For asparagus SDF, the results of the colorimetric approaches using cuvettes (22.02 ± 0.10) and 24-well plates (19.77 ± 0.97) as well as the results of the colorimetric 24-well plates approach and the approach using an ammonia electrode (22.03 ± 0.34) differed significantly. For wild rice SDF, results obtained from classical titration (23.01 ± 0.05) and from the colorimetric approach using cuvettes (25.07 ± 0.46) as well as those from the colorimetric assays using either cuvettes or 24-well plates (23.06 ± 0.91) differed. For pear IDF, residual protein contents obtained from titration (6.20 ± 0.05) and from the colorimetric assay using cuvettes (5.39 ± 0.18) turned out to be different. Although these differences are statistically significant they appear not to be of major relevance, because the deviations are in an acceptable range.

**Table 1:** Protein contents of preparative dietary fiber preparations in percent determined by different detection methods after Kjeldahl digestion.

	**(1) Titration**	**(2) Cuvettes**	**(3) 24-Well plates**	**(4) Electrode**
asparagus IDF	9.44 ± 0.15	9.12 ± 0.17	9.36 ± 0.70	9.26 ± 0.13
asparagus SDF	21.14 ± 0.06^ab^	22.02 ± 0.10^a^	19.77 ± 0.97^b^	22.03 ± 0.34^a^
wild rice IDF	50.22 ± 0.35	52.08 ± 1.63	51.36 ± 1.05	49.51 ± 0.69
wild rice SDF	23.01 ± 0.05^a^	25.07 ± 0.46^b^	23.06 ± 0.91^a^	23.63 ± 0.45^ab^
buckwheat IDF	21.72 ± 0.27	20.71 ± 0.43	21.07 ± 1.03	21.69 ± 0.49
buckwheat SDF	35.95 ± 0.30	37.64 ± 0.63	34.23 ± 1.96	35.95 ± 0.43
pear IDF	6.20 ± 0.05^a^	5.39 ± 0.18^b^	5.96 ± 0.25^ab^	5.50 ± 0.25^ab^
pear SDF	13.83 ± 0.03	12.45 ± 0.18	13.00 ± 1.12	13.03 ± 0.16
wheat IDF	14.54 ± 0.04	14.42 ± 0.20	13.75 ± 0.04	14.59 ± 0.46
wheat SDF	18.74 ± 0.08	19.16 ± 0.08	18.60 ± 0.90	18.30 ± 0.49

*The tested methods comprise a (1) classical titration with diluted HCl after steam distillation, the spectrophotometric method according to Willis et al. (8) in (2) cuvettes or in (3) 24-well plates, and the determination using an (4) ammonia electrode. IDF, insoluble dietary fiber; SDF, soluble dietary fiber. The data was statistically analyzed for each sample individually using one-way ANOVA and Tukey's test (α = 0.01). Values of a specific sample with the same letter are in the same group*.

According to these results and despite the slightly higher standard deviations as compared to the conventional titration method we found the application of an ammonia electrode to be the most time-efficient and straightforward method to detect ammonia in Kjeldahl digests of DF preparations. Also, this method requires lower sample amounts than the conventional titration method and can be performed from digests prepared in micro Kjeldahl digestion units.

### Amino Acid Profiles of Residual Protein in Dietary Fiber Preparations

The amino acid profile of DF preparations was determined for two reasons. Firstly, application of the widely used Kjeldahl conversion factor of 6.25 is only weakly supported by literature and is presumably dating back to the Nineteenth century (9). It is based on the assumption that the average nitrogen content of a protein is about 16%. However, the nitrogen content of proteins certainly depends on their amino acid profiles. Whereas, amino acids that are rich in nitrogen such as arginine (32.1% N), histidine (27.1% N), asparagine (21.2% N), and glycine (18.6% N) would lower this factor, amino acids that are poor in nitrogen such as tyrosine (7.7%) and phenylalanine (8.5%) would increase it. Jones pointed out this problem already in 1941 and provided specific nitrogen conversion factors for different proteins (23). Secondly and more importantly, we were interested whether the residual protein content of the DF preparations is predominantly due to protease resistant plant cell wall structural proteins.

Here, we used materials, eluents, and reagents that make the amino acid profiling approach following AQC derivatization independent of commercial kit suppliers as previously shown by Gwatidzo et al. (17). A drawback of the AQC derivatization technique is that it does not result in stable derivatives of tryptophan (24). In addition, tryptophan is substantially degraded during acid hydrolysis (25). Therefore, tryptophan is not included into the amino acid profiles shown here. Also, cysteine and methionine are partly degraded during acid hydrolysis, and the amide groups of glutamine and asparagine are converted into the corresponding carboxyl groups, thereby forming glutamic acid and aspartic acid (25). In order to establish a standard method for the determination of amino acids, an official method of the European Commission was established. In this method, the sulfur containing amino acids are oxidized prior to quantitation and detected as the stabile derivatives cysteic acid and methionine sulfone. However, this official method does not determine tryptophan, asparagine, or glutamine in a reliable manner, too (26).

**Figure 2:**
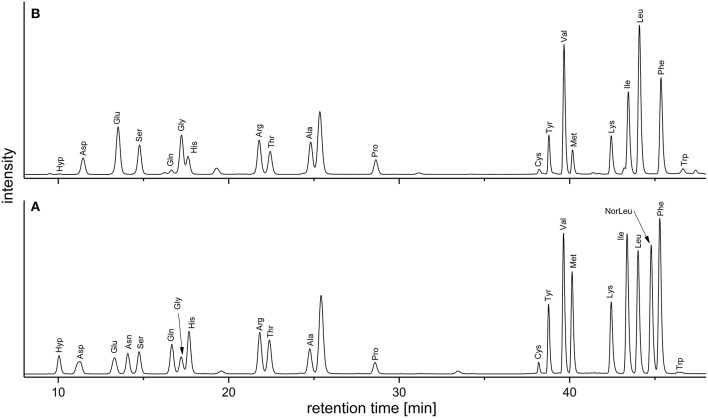
Chromatograms (fluorescence detection; 250/395 nm) of the AQC-derivatives obtained from **(A)** an amino acid standard mixture and **(B)** the acidic hydrolysate of a wheat IDF sample [without the internal standard norleucine (NorLeu)]. AQC, 6-aminoquinolyl-*N*-hydroxysuccinimidyl carbamate; IDF, insoluble dietary fiber.

Keeping the downsides of the AQC method in mind, we deemed it acceptable as a quick and easy approach to routinely screen the amino acid profiles of DF preparations. In addition, and in contrast to the official EC-method the AQC method can be used to detect hydroxyproline, an important marker for structural proteins. Figure 2 demonstrates the separation of the analytes in a standard mixture and in a sample solution. Table 2 shows the amino acid profiles of the DF preparations obtained by using the preparative approach described in section Preparative Isolation of Soluble and Insoluble Dietary Fiber.

**Table 2:** Amino acid profile of dietary fiber preparations that were obtained by the preparative approach.

	**Pear IDF**	**Pear SDF**	**Asparagus IDF**	**Asparagus SDF**	**Buckwheat IDF**	**Buckwheat SDF**	**Wild rice IDF**	**Wild rice SDF**	**Wheat IDF**	**Wheat SDF**
Hyp	0.82 ± 0.05	0.36 ± 0.02	4.55 ± 0.27	1.95 ± 0.16	0.30 ± 0.04	0.08 ± 0.02	0.28 ± 0.03	0.52 ± 0.00	0.12 ± 0.00	0.23 ± 0.03
Asp	10.83 ± 0.70	13.70 ± 0.97	17.42 ± 1.06	24.98 ± 2.05	10.56 ± 0.83	12.10 ± 1.02	10.93 ± 0.40	17.90 ± 0.04	13.34 ± 0.10	11.22 ± 0.39
Glu	9.60 ± 0.43	8.25 ± 0.41	11.98 ± 0.50	10.87 ± 0.55	14.37 ± 0.89	17.33 ± 1.13	12.51 ± 0.22	16.48 ± 0.03	14.85 ± 0.16	16.46 ± 0.50
Ser	6.40 ± 0.39	11.01 ± 0.43	6.42 ± 0.27	8.15 ± 0.50	5.80 ± 0.16	7.04 ± 0.02	5.34 ± 0.13	5.77 ± 0.02	5.42 ± 0.02	8.29 ± 0.09
Gln	1.62 ± 0.10	0.69 ± 0.09	1.62 ± 0.53	2.02 ± 1.15	0.78 ± 0.36	0.49 ± 0.35	1.15 ± 0.09	1.94 ± 0.01	0.46 ± 0.01	0.70 ± 0.43
Gly	8.95 ± 0.25	7.94 ± 0.35	8.79 ± 0.39	8.09 ± 0.31	9.44 ± 0.32	9.47 ± 0.18	7.86 ± 0.12	6.07 ± 0.01	7.34 ± 0.05	9.83 ± 0.26
His	2.30 ± 0.03	1.21 ± 0.08	2.36 ± 0.25	3.02 ± 0.25	2.01 ± 0.04	2.08 ± 0.02	2.45 ± 0.04	2.36 ± 0.01	2.03 ± 0.02	3.38 ± 0.21
Arg	3.24 ± 0.21	2.61 ± 0.10	3.65 ± 0.18	3.90 ± 0.32	6.15 ± 0.16	8.16 ± 0.21	5.03 ± 0.05	2.66 ± 0.02	9.19 ± 0.05	5.65 ± 0.09
Thr	5.21 ± 0.14	10.12 ± 0.33	3.97 ± 0.14	5.07 ± 0.28	4.23 ± 0.10	4.50 ± 0.07	4.20 ± 0.07	3.91 ± 0.02	3.87 ± 0.02	5.69 ± 0.04
Ala	9.32 ± 0.11	11.36 ± 0.25	7.96 ± 0.14	7.67 ± 0.05	7.62 ± 0.07	6.14 ± 0.02	8.86 ± 0.06	5.51 ± 0.01	7.24 ± 0.01	7.68 ± 0.09
Pro	5.44 ± 0.29	4.04 ± 0.06	4.81 ± 0.11	2.40 ± 0.10	4.48 ± 0.11	3.15 ± 0.10	4.60 ± 0.02	4.82 ± 0.00	6.16 ± 0.08	4.54 ± 0.03
Cys	2.32 ± 0.13	1.20 ± 0.33	1.61 ± 0.27	2.46 ± 0.15	3.10 ± 0.32	5.38 ± 1.53	2.69 ± 0.14	3.18 ± 0.01	2.41 ± 0.02	5.02 ± 0.20
Tyr	2.29 ± 0.14	3.55 ± 0.13	2.14 ± 0.11	2.30 ± 0.29	2.10 ± 0.10	2.23 ± 0.02	3.20 ± 0.04	1.78 ± 0.01	3.02 ± 0.02	2.77 ± 0.25
Val	6.96 ± 0.13	6.21 ± 0.07	5.01 ± 0.11	3.34 ± 0.09	6.40 ± 0.10	4.37 ± 0.09	7.02 ± 0.03	7.70 ± 0.01	5.21 ± 0.04	4.40 ± 0.03
Met	0.42 ± 0.06	0.13 ± 0.03	0.46 ± 0.08	0.38 ± 0.10	1.42 ± 0.12	1.11 ± 0.03	2.59 ± 0.14	0.40 ± 0.00	1.59 ± 0.01	0.68 ± 0.03
Lys	6.41 ± 0.45	3.71 ± 0.12	5.30 ± 0.32	7.16 ± 0.63	5.16 ± 0.23	5.90 ± 0.67	4.51 ± 0.13	4.80 ± 0.03	3.50 ± 0.02	4.67 ± 0.24
Ile	5.22 ± 0.21	3.89 ± 0.06	3.56 ± 0.05	1.69 ± 0.25	4.53 ± 0.17	2.87 ± 0.10	5.40 ± 0.13	6.06 ± 0.04	4.08 ± 0.03	2.61 ± 0.09
Leu	8.84 ± 0.37	6.95 ± 0.09	5.66 ± 0.08	3.07 ± 0.05	7.79 ± 0.84	5.13 ± 0.66	7.65 ± 0.03	7.01 ± 0.02	6.96 ± 0.04	4.36 ± 0.04
Phe	3.79 ± 0.32	3.07 ± 0.04	2.73 ± 0.22	1.50 ± 0.16	3.76 ± 0.15	2.48 ± 0.02	3.75 ± 0.05	1.15 ± 0.00	3.23 ± 0.03	1.80 ± 0.18

*IDF, insoluble dietary fiber; SDF, soluble dietary fiber*.

Aspartic acid, glutamic acid, glycine, alanine, leucine, and valine are the dominant constituent amino acids of residual protein in all DF preparations. Amino acids that are common amino acids of structural proteins of plants (found in repeated sequence motifs also containing additional amino acids) are dominated by proline and glycine as their proportions range from 2.40 to 6.16 and from 6.07 to 9.83%, respectively, whereas the hydroxyproline portion was almost always below 1%. Differently, the hydroxyproline proportion in asparagus IDF and SDF preparations was 4.55 and 1.95%, respectively. These results are consistent with earlier studies of Minero-Amador et al. (27) who assigned the polymeric hydroxyproline contents of asparagus samples to hydroxyproline-rich glycoproteins of the primary cell wall.

We and others assumed that the residual protein that is resistant to the enzymatic DF isolation procedure consists mainly of (insoluble) structural proteins, because they are often highly glycosylated and/or embedded via ionic or covalent bonds in a matrix made up of other plant cell wall polymers. Hence, proteases that are used in the DF isolation procedure may be (sterically) hindered from degrading them. As mentioned before, structural proteins of plants are characterized by their high contents of the amino acids glycine, proline, and/or hydroxyproline (15). Thus, the amino acid profiles of the residual protein as analyzed here (quite diverse and dominated by many other amino acids) do not support our previous hypothesis at this time. However, more in-depth studies are required to understand the fate of structural proteins during the DF isolation procedure and to identify the constituent proteins of the residual protein fraction.

Additionally, we used our screening method to compare the residual protein amino acid profiles of IDF preparations that were obtained by using either the official analytical method according to AOAC 2009.01 (described in section Analytical Determination/Isolation of Dietary Fiber; here we used subtilisin A (subtilisin Carlsberg); the protocol does not ask for a specific protease, but subtilisin A has traditionally been used) or the “in-house” preparative method (the protease product Alcalase 2.5 L (protease activity appears to be primarily due to subtilisin A) was used) as different protease preparations and pH conditions were used. Generally, the amino acid profiles are not identical, but comparable and often only differ in details, but not substantially (Tables 2, 3). However, the portions of glycine and alanine are higher in the IDF preparations obtained by using the analytical approach, which is compensated for by lower values for lysine and isoleucine. Despite these differences it can be concluded that different enzyme preparations (both of which were obtained from *Bacillus licheniformis*; however, different degrees of purity are assumed) and pH conditions applied during the isolation procedure do not result in substantially different conclusions about the residual protein composition of DF preparations.

**Table 3:** Amino acid profiles of insoluble dietary fiber (IDF) preparations that were obtained applying the official analytical AOAC method (2009.01) according to McCleary et al. (7).

	**Pear IDF**	**Buckwheat IDF**	**Wild rice IDF**	**Wheat IDF**
Hyp	0.82 ± 0.03	0.51 ± 0.02	0.35 ± 0.01	0.25 ± 0.01
Asp	10.79 ± 0.07	9.74 ± 0.06	9.93 ± 0.10	8.59 ± 0.07
Glu	10.46 ± 0.07	13.24 ± 0.03	11.34 ± 0.07	16.68 ± 0.16
Ser	7.43 ± 0.01	5.54 ± 0.02	5.82 ± 0.02	6.44 ± 0.09
Gln	2.77 ± 0.02	0.80 ± 0.18	1.25 ± 0.01	1.32 ± 0.01
Gly	12.34 ± 0.03	10.35 ± 0.21	10.46 ± 0.08	13.31 ± 0.09
His	2.12 ± 0.03	2.18 ± 0.01	2.52 ± 0.04	2.81 ± 0.02
Arg	3.23 ± 0.02	4.14 ± 0.01	4.51 ± 0.02	4.73 ± 0.05
Thr	4.87 ± 0.09	4.21 ± 0.01	4.21 ± 0.07	3.84 ± 0.01
Ala	11.28 ± 0.05	12.17 ± 0.02	10.37 ± 0.08	9.48 ± 0.08
Pro	4.99 ± 0.06	4.57 ± 0.07	3.89 ± 0.01	6.42 ± 0.13
Cys	3.11 ± 0.60	2.96 ± 0.16	3.43 ± 0.51	3.28 ± 0.90
Tyr	2.14 ± 0.02	2.52 ± 0.02	3.25 ± 0.06	2.27 ± 0.02
Val	5.69 ± 0.06	6.24 ± 0.04	6.48 ± 0.07	4.65 ± 0.05
Met	0.72 ± 0.01	1.24 ± 0.01	2.02 ± 0.01	1.08 ± 0.01
Lys	3.01 ± 0.02	3.85 ± 0.01	3.79 ± 0.05	3.09 ± 0.02
Ile	3.87 ± 0.04	4.27 ± 0.03	4.51 ± 0.01	2.90 ± 0.05
Leu	6.82 ± 0.06	7.65 ± 0.04	7.84 ± 0.05	5.64 ± 0.09
Phe	3.54 ± 0.05	3.81 ± 0.03	4.02 ± 0.04	3.21 ± 0.05

By using amino acid profiles, it is theoretically possible to calculate exact Kjeldahl conversion factors. In practice, however, several limitations exist (9, 10). For example, due to the aforementioned hydrolysis of glutamine and asparagine to glutamic acid and aspartic acid during the acid hydrolysis it is only possible to calculate a range that comprises the accurate value. Table 4 shows the ranges of Kjeldahl conversion factors calculated by using the amino acid profiles displayed in Table 2. The lower limits assume that the total measured glutamic acid and aspartic acid content were initially the corresponding amides glutamine and asparagine. The upper limits are based on the contrary assumption. These values indicate that the general conversion factor of 6.25 might be too high for some of the chosen samples. Only the upper limits of the SDF samples of pear (5.98–6.32) and wild rice (5.82–6.50) exceeded 6.25. However, interpretation of these data needs to consider that cysteine, methionine, and tryptophan, which are also partly degraded during the acid hydrolysis, would increase these values as their amino acid specific conversion factors are 8.65, 10.66, and 7.29, respectively.

**Table 4:** Calculated ranges of Kjeldahl conversion factors according to Formula 1 based on the amino acid profiles of dietary fiber preparations obtained by the preparative approach.

	**Lower limit**	**Upper limit**
IDF pear	5.76	6.13
SDF pear	5.98	6.32
IDF asparagus	5.74	6.21
SDF asparagus	5.67	6.09
IDF buckwheat	5.52	6.05
SDF buckwheat	5.30	5.90
IDF wild rice	5.69	6.18
SDF wild rice	5.82	6.50
IDF wheat	5.44	5.98
SDF wheat	5.38	5.96

*Due to the partial conversion of Gln and Asn to Glu and Asp during the acid hydrolysis ranges are given instead of single numbers (see text). IDF, insoluble dietary fiber; SDF, soluble dietary fiber*.

These data demonstrate again that the exact determination of Kjeldahl conversion factors is challenging; determination of individual conversion factors is always a compromise between accuracy and practicability. However, with all limitations described, our data show that the conversion factors for different fiber preparations vary and that using a single conversion factor is certainly a source of errors in the determination of DF contents. As protein correction unquestionably has an impact on analyzed dietary fiber contents approaches to counter this inaccuracy in dietary fiber analysis need to be explored. The most practicable approach would be to maximally hydrolyze protein during the fiber isolation procedure, thus minimizing the effect of an inaccurate correction factor. Traditionally, subtilisin A (subtilisin Carlsberg) has been used in most dietary fiber isolation procedures as it is a serine endopeptidase of broad specificity (28). Whether other proteases (either applied individually or in combination) are more suitable to hydrolyze protein in the dietary fiber isolation process will require some attention in future method improvements. Another path would be to suggest individual correction factors for dietary fiber preparations of different origin. However, this approach appears to be extremely laborious; also, the technological history of a sample might have an impact on the protein digestibility during the fiber isolation process.

## Conclusion

In this study we were able to demonstrate the applicability of an ammonia electrode for fast, reliable and safe detection of ammonia after Kjeldahl digestion of DF preparations. This approach results in similar nitrogen contents of the Kjeldahl digests as the common titrimetric method as well as a spectrophotometric approach. In addition, we adapted the AQC derivatization approach to acidic hydrolysates of residual protein that is contained in DF preparations after using the enzymatic gravimetric procedure. The amino acid profiles obtained do not support our previous hypothesis that residual protein is mostly made up of structural proteins. The amino acid profiles also suggest that the commonly used Kjeldahl conversion factor of 6.25 might be too high for the determination of residual protein in DF preparations. Although the calculated factors contain some experimental uncertainties they already emphasize the need to monitor Kjeldahl factors in a wide range of DF preparations more closely and to develop strategies to minimize this source of error in dietary fiber analysis.

## Author Contributions

FU, DW, and MB designed the research. PM conducted the experiments, FU, PM, and AH analyzed data and results. FU and MB wrote the manuscript. All authors approved the final version of the manuscript.

### Conflict of Interest Statement

The authors declare that the research was conducted in the absence of any commercial or financial relationships that could be construed as a potential conflict of interest.

## Abbreviations

AQC: 6-aminoquinolyl-*N*-hydroxysuccinimidyl carbamate
DF: dietary fiber
IDF: insoluble dietary fiber
NorLeu: norleucine
SDF: soluble dietary fiber.
